# Correlation of Serotype-Specific Dengue Virus Infection with Clinical Manifestations

**DOI:** 10.1371/journal.pntd.0001638

**Published:** 2012-05-01

**Authors:** Eric S. Halsey, Morgan A. Marks, Eduardo Gotuzzo, Victor Fiestas, Luis Suarez, Jorge Vargas, Nicolas Aguayo, Cesar Madrid, Carlos Vimos, Tadeusz J. Kochel, V. Alberto Laguna-Torres

**Affiliations:** 1 United States Naval Medical Research Unit Six, Lima, Perú; 2 Department of Epidemiology, Johns Hopkins Bloomberg School of Public Health, Baltimore, Maryland, United States of America; 3 Universidad Peruana Cayetano Heredia, Lima, Peru; 4 Instituto Nacional de Salud, Ministerio de Salud, Lima, Peru; 5 Direccion General de Epidemiologia, Ministerio de Salud, Lima, Peru; 6 Centro Nacional de Enfermedades Tropicales, Ministerio de Salud, Santa Cruz, Bolivia; 7 Organización No Gubernamental Rayos de Sol, Asunción, Paraguay; 8 Hospital Naval, Guayaquil, Ecuador; 9 Direccion Departamental de Pastaza, Ministerio de Salud del Ecuador, Puyo, Ecuador; Centre for Cellular and Molecular Biology (CCMB), India

## Abstract

**Background:**

Disease caused by the dengue virus (DENV) is a significant cause of morbidity throughout the world. Although prior research has focused on the association of specific DENV serotypes (DENV-1, DENV-2, DENV-3, and DENV-4) with the development of severe outcomes such as dengue hemorrhagic fever and dengue shock syndrome, relatively little work has correlated other clinical manifestations with a particular DENV serotype. The goal of this study was to estimate and compare the prevalence of non-hemorrhagic clinical manifestations of DENV infection by serotype.

**Methodology and Principal Findings:**

Between the years 2005–2010, individuals with febrile disease from Peru, Bolivia, Ecuador, and Paraguay were enrolled in an outpatient passive surveillance study. Detailed information regarding clinical signs and symptoms, as well as demographic information, was collected. DENV infection was confirmed in patient sera with polyclonal antibodies in a culture-based immunofluorescence assay, and the infecting serotype was determined by serotype-specific monoclonal antibodies. Differences in the prevalence of individual and organ-system manifestations were compared across DENV serotypes. One thousand seven hundred and sixteen individuals were identified as being infected with DENV-1 (39.8%), DENV-2 (4.3%), DENV-3 (41.5%), or DENV-4 (14.4%). When all four DENV serotypes were compared with each other, individuals infected with DENV-3 had a higher prevalence of musculoskeletal and gastrointestinal manifestations, and individuals infected with DENV-4 had a higher prevalence of respiratory and cutaneous manifestations.

**Conclusions/Significance:**

Specific clinical manifestations, as well as groups of clinical manifestations, are often overrepresented by an individual DENV serotype.

## Author Summary

Dengue virus (DENV) causes disease in millions of people annually and disproportionately affects those in the developing world. DENVs may be divided into four serotypes (DENV-1, DENV-2, DENV-3, and DENV-4) and a geographical region may be affected by one or more DENV serotypes simultaneously. Infection with DENV may cause life-threatening disease such as dengue hemorrhagic fever (DHF) or dengue shock syndrome (DSS), but more often causes less severe manifestations affecting a wide range of organs. Although many previous reports have explored the role of the different DENV serotypes in the development of severe manifestations, little attention has focused on the relative role of each DENV serotype in the development of cutaneous, respiratory, gastrointestinal, musculoskeletal, and neurological manifestations. We recruited a large group of participants from four countries in South America to compare the prevalence of more than 30 manifestations among the four different DENV serotypes. We found that certain DENV serotypes were often associated with a higher prevalence of a certain manifestation (e.g., DENV-3 and diarrhea) or manifestation group (e.g., DENV-4 and cutaneous manifestations).

## Introduction

The world is experiencing a rapid increase in dengue virus (DENV) infections with an estimated 50 million people infected annually, and nearly two-fifths of the world's population is living in areas considered high-risk for infection [1]. DENVs are divided into four antigenically distinct serotypes (DENV-1, DENV-2, DENV-3, and DENV-4), and human infection may be asymptomatic or manifest as dengue fever, dengue hemorrhagic fever (DHF), or dengue shock syndrome (DSS). Despite widespread eradication of the mosquito vector *Aedes aegypti* in the 1950s, the continent of South America has once again become an epicenter of this spreading epidemic with nearly a five-fold increase in the incidence of detectable dengue infection over the last three decades and a concomitant expansion of the number of circulating serotypes [2]. For instance, Bolivia experienced its first re-emergent dengue fever case in 1987, followed by Paraguay and Ecuador in 1988, and Peru in 1990 [3]. At that time, only DENV-1 was present. Today, all four of these countries have experienced the introduction of multiple serotypes.

The most common form of symptomatic DENV infection, dengue fever, often presents with fever, headache, and severe bone and joint pains [4]. DENV infection also commonly affects a wide array of organ systems, including the dermatologic, neurologic, respiratory, and gastrointestinal systems [5]. Less commonly described manifestations include those affecting the cardiac [6], lymphoreticular [6], renal [6], and ocular [7] systems.

Risk factors for the severe manifestations of DHF and DSS have been attributed to a multitude of factors, including secondary versus primary infection [8], specific serotype [9] or genotype [10], gender [11], and age [12]. While much attention has focused on the factors causing severe and hemorrhagic disease, much less has been dedicated to comparing differences in specific clinical manifestations by DENV serotype. Only a handful of reports [13], [14], [15], [16], [17], [18], [19], [20], [21], [22] (**Table 1**) have used serotype-confirmed DENV infection to compare non-hemorrhagic clinical manifestations between serotypes, and there is heterogeneity across these studies. Many of these studies lacked a large sample size, restricted their analyses to inpatients, did not examine all four DENV serotypes, or did not recruit both children and adults.

**Table 1:** Studies with serotype-confirmed DENV infections that compared non-hemorrhagic clinical manifestations between multiple DENV serotypes.

Author	Publication Year	Country	# of patients	Patient age	Emergency room, In-patient, or Out-patient	Serotypes	Method of confirmation	Statistically significant findings (DENV Serotype)
Balmaseda [18]	2006	Nicaragua	150*	Age <15	In-patient	1,2	Culture, PCR	None
Chan [14]	2009	Taiwan	294	Adults	In-patient	2,3	PCR	Rash (not petechial): 3>2
								Ascites: 3>2
								Cough, nasal stuffiness, rhinorrhea, or sore throat: 3>2
								Myalgia: 3>2
								Bone pain: 2>3 (p = .051)
Chuang [13]	2008	Hong Kong	126	All ages	In-patient	1,2,3,4	PCR	None
Fried [19]	2010	Thailand	458	Children	In-patient and out-patient	1,2,3,4	PCR	Ascites: 2> all other serotypes
								Pleural effusion index: 2>1
Kalayanarooj [20]	2000	Thailand	2398	Children	In-patient	1,2,3,4	Culture, PCR	Hepatomegaly: (2,3)>(1,4)
Kumaria [22]	2010	India	80	Not specified	In-patient and out-patient	1,2,3,4	PCR	Anorexia: 2>(1, 4)>3
								Abdominal pain: 2>(1,4)>3
								Hepatomegaly: 2>1,3,4
Mostorino [16]	2002	Peru	236	All ages	In-patient and out-patient	1,2,3	Culture	Rash (not petechial): 1>2>3
								Sore throat: 1>2>3
								Nasal congestion: 2>1>3
								Retro-ocular pain: 1>2>3
								Body pain: 1>2>3
								Bone pain: 2>1>3
								Chills: 1>2>3
								Inappetite:2>1>3
Sumarmo [15]	1983	Indonesia	30	All ages	In-patient**	1,2,3,4	Culture	None
Thai [21]	2010	Vietnam	86	All ages	Out-patient	1,2,3,4	PCR	None
Thomas [17]	2008	Martinique	146	Age >15	Emergency room	2,4	PCR	GI signs: 2>4

*: Serotype confirmation was performed on 150 of 1297 total participants.

**: Only analyzed fatal cases.

The goal of the current cross-sectional study was to estimate and compare the prevalence of specific clinical signs and symptoms by DENV serotype among a large number of individuals participating in a passive clinic-based surveillance system for febrile illness across western South America.

## Methods

### Study Population

**Figure 1:**
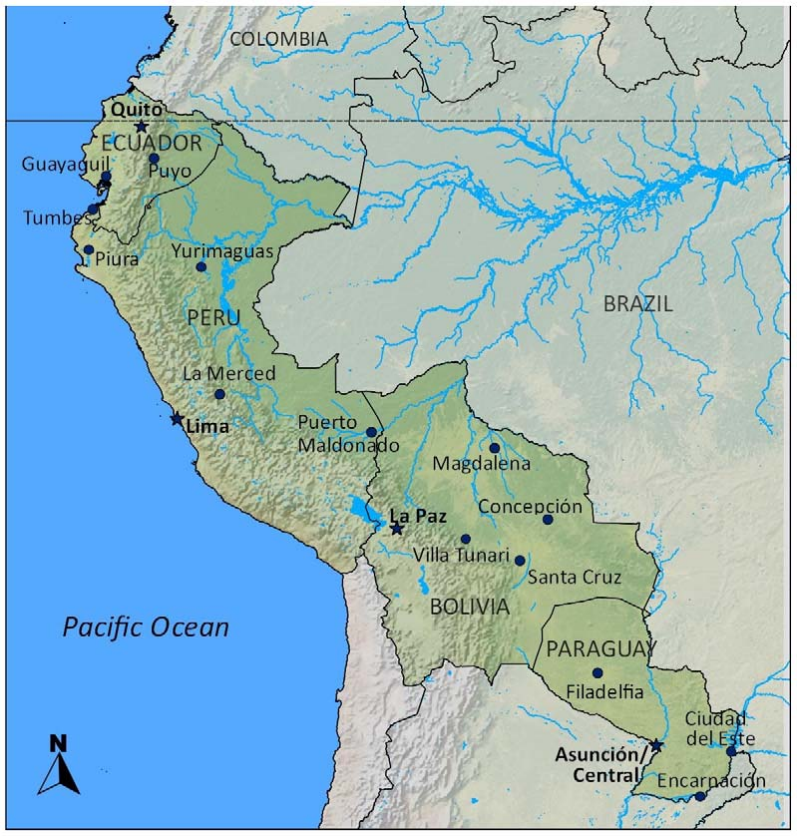
Location of participant recruitment sites.

In 2000, the Naval Medical Research Center Detachment (NMRCD) initiated an outpatient passive surveillance system to detect acute febrile disease in Peru and Bolivia [23]. In 2001 and 2005, study sites in Ecuador and Paraguay were added, respectively. All recruitment was performed at outpatient clinics (**Figure 1**) by local doctors trained in the protocol and recruitment specifications. A site utilizing medical technicians (Iquitos, Peru) instead of medical doctors for data collection was excluded from our analysis. Participants eligible for recruitment: were five years of age or older, had a temperature ≥38°C, had no obvious focus of infection, and were able to sign a consent form. Participants younger than age 18 provided written assent following written consent from their parent or guardian. Participants included in this study were recruited between the dates of January 1, 2005 and August 20, 2010.

### Ethics Statement

Study protocols (NMRCD.2000.0006 [Peru], NMRCD.2000.0008 [Bolivia], NMRCD.2001. 0002 [Ecuador], and NMRCD.2005.0008 [Paraguay]) were approved by the Naval Medical Research Center Institutional Review Board (Bethesda, MD) in compliance with all U.S. Federal regulations governing the protection of human subjects. In addition, study protocols were reviewed and approved by health authorities in Peru (Dirección General de Epidemióloga and Instituto Nacional de Salud), Bolivia (Servicio Departamental de Salud, Santa Cruz, and Colegio Médico de Santa Cruz), Ecuador (Ministerio de Salud Publica and Escuela de Sanidad Naval in Guayaquil), and Paraguay (Ministerio de Salud y Bienestar Social and Comité de Ética de Asociacion Rayos de Sol).

### Data and Sample Collection

A complete history and physical examination was performed on every participant by a study doctor at each outpatient site. Each doctor was provided with the same data collection form that possessed an extensive list of signs and symptoms. The form also contained basic demographic data such as age, gender, and town of residence, as well as day of illness onset. All information used in this study was collected at the initial visit. A blood sample was obtained by venipuncture from the arm, using standard methods.

### Laboratory Methods

DENV isolation was performed on acute serum samples taken from patients seven days or fewer following the onset of illness on the first day they sought medical care in our study. Sera were inoculated on *Aedes albopictus* C6/36 and African green monkey kidney (Vero) cells. Upon observation of cytopathic effect or after ten days, the cells were collected by centrifugation and examined by indirect immunofluorescence assay (IFA) using screening dengue polyclonal antisera. To identify the specific DENV serotype, serotype-specific monoclonal antibodies were generated using hybridomas obtained from the Centers of Disease Control and Prevention. On the acute sera, IgM and IgG titers were measured using a previously described enzyme-linked immunosorbent assay [23]. IgM/IgG ratios were then calculated and compared with a ratio known to discriminate between primary and secondary infections, similar to an approach used by others [24].

### Clinical Definitions

Participants with serotype-specific dengue infection were defined as having sera with DENV confirmed by culture/IFA. In order to take a broader look at how often certain groups of manifestations were affected by specific serotypes, the following clinical manifestation groups were constructed: “Constitutional manifestations” was defined as having malaise, prostration, headache, or retro-orbital pain; “Respiratory manifestations” was defined as having a cough, dyspnea, rhinorrhea, pharyngeal congestion, wheezing, cyanosis, or rhonchi; “Gastrointestinal manifestations” was defined as having abdominal pain, abdominal distension, diarrhea, nausea, vomiting, ascites, hepatomegaly, splenomegaly, or jaundice; “Musculoskeletal manifestations” was defined as having bone pain, myalgia, or joint pain (a combined endpoint of arthritis, arthalgia, and incapacity of joint function); “Cutaneous manifestations” was defined as having a maculopapular rash, central erythema, distal erythema, facial erythema, vesicles, or subcutaneous nodules; “Neurological manifestations” was defined as having seizures, neck stiffness, impaired mental status, or a focal neurological deficit. For a participant to be noted as being affected by a certain manifestation category, they needed to have at least one specific manifestation from that category. Vital signs were not collected.

### Statistical Analysis

The objective of this analysis was to compare the prevalence of clinical manifestations across individuals with the four different DENV serotypes. Overall, there were 1938 individuals presenting with febrile illness at clinics in western South America and diagnosed with one of four DENV serotypes. Individuals with missing information on sex (n = 1) and date of illness (n = 5) were excluded from the analysis. There were 216 individuals with missing data on one or more sign or symptom. These individuals did not differ from the remaining individuals with respect to age, sex, location, or DENV serotype and therefore were excluded from the analysis. Because differences in demographic factors could confound the primary comparisons of the prevalence of manifestations between DENV serotypes, demographic variables such as age, sex, city, and year of diagnosis were assessed. Age was categorized into deciles of ≤10 years, 11–20 years, 21–30 years, 31–40 years, 41–50 years, 51–60 years, >60 years. Time since illness onset was based on the interval between the clinic visit and a participant's self-reported first occurrence of febrile illness. This measure was defined in days and treated as continuous in the analysis. Country and city of residence was categorized as Peru (La Merced, Puerto Maldonado, Piura, Tumbes, Yurimaguas), Bolivia (Concepcion, Magdalena, Santa Cruz, Villa Tunari), Ecuador (Guayaquil, Puyo), and Paraguay (Asuncion, Central, Ciudad del Este, Encarnacion, Filadelfia). Year of diagnosis was treated as continuous.

Contingency tables were created to assess the prevalence of clinical manifestations and demographic variables with serotype-specific DENV infection. Differences in the distribution of demographic variables across DENV serotype were evaluated using Pearson's chi-square statistic or Wilcoxon Rank Sum test for categorical or continuous data, respectively.

Given the high prevalence of clinical manifestations in this study population, odds ratios as a measure of association calculated using logistic regression could have overestimated the strength of the association [25]. Therefore, prevalence ratios (PR) were estimated using Poisson regression with robust variance [26]. Based on the laboratory-confirmed DENV serotype, a dummy variable was created that classified each individual as having been infected with a given DENV serotype versus infection with another DENV serotype (e.g., DENV-1 vs. non-DENV-1). All models compared the prevalence of a given manifestation among individuals with a given serotype-specific DENV infection against those infected with the other three DENV serotypes and were adjusted by age, sex, city, days of illness at presentation, DENV infection status (primary versus secondary), and year of diagnosis. Lastly, given the large number of comparisons across multiple clinical manifestations within a given group of individuals infected with a specific DENV serotype, a more conservative p-value estimate of 0.005 was used as a cut-off for statistical significance based on the Bonferroni correction for multiple comparisons. All analyses were performed using STATA 11.0 (STATACORP; College Station, TX).

## Results

### Distribution of Demographic Factors and DENV Serotype

Between January 1, 2005, and August, 20, 2010, 1, 716 individuals were identified as infected with a DENV with the following breakdown: DENV-1, 39.8%; DENV- 2, 4.3%; DENV- 3, 41.5%; or DENV-4, 14.4% (**Table 2**). The median age of the sample was 29 years (IQR: 20, 41). While there was no significant difference in the mean age by DENV serotype, individuals with DENV-1 were generally younger (<20 years of age). A higher proportion of men were diagnosed with DENV-2.

**Table 2:** Association of demographic variables and dengue serotype.

	TotalN(%)(N = 1,716)	DENV-1(n = 683) (39.8%)	DENV-2(n = 74) (4.3%)	DENV-3(n = 712) (41.5%)	DENV-4(n = 247) (14.4%)
		N(%)	N(%)	N(%)	N(%)
Age (by decile):					
≤10	74 (4.3)	42 (6.2)	2 (2.7)	19 (2.7)	11 (4.5)
11–20	390 (22.7)	171 (25.0)	14 (18.9)	150 (21.1)	55 (22.3)
21–30	466 (27.2)	193 (28.3)	20 (27.0)	196 (27.5)	57 (23.1)
31–40	334 (19.5)	110 (16.1)	17 (22.9)	154 (21.6)	53 (21.5)
41–50	271 (15.8)	98 (14.3)	15 (20.3)	120 (16.9)	38 (15.4)
51–60	127 (7.4)	43 (6.3)	5 (6.8)	53 (7.4)	26 (10.5)
>60	54 (3.1)	26 (3.8)	1 (1.4)	20 (2.8)	7 (2.7)
Sex:					
Male	833 (48.5)	320 (46.9)	47 (63.5)	352 (49.4)	114 (46.2)
Female	883 (51.5)	363 (53.1)	27 (36.5)	360 (50.6)	133 (53.8)
Time since symptom onset (days):					
0	79 (4.6)	26 (3.8)	4 (5.4)	40 (5.6)	9 (3.7)
1	373 (21.7)	146 (21.4)	16 (21.6)	165 (23.2)	46 (18.7)
2	469 (27.3)	184 (26.9)	21 (28.3)	190 (26.8)	74 (29.9)
3	392 (22.8)	160 (23.4)	14 (18.9)	149 (20.9)	69 (27.9)
4	189 (11.1)	77 (11.3)	7 (9.5)	78 (10.9)	27 (10.9)
5	91 (5.3)	32 (4.7)	5 (6.7)	45 (6.4)	9 (3.6)
6	41 (2.4)	16 (2.4)	3 (4.1)	17 (2.4)	5 (2.1)
7	58 (3.4)	27 (3.9)	3 (4.1)	21 (2.9)	7 (2.8)
Unknown	24 (1.4)	15 (2.2)	1 (1.4)	7 (0.9)	1 (0.4)
Infection type:					
Primary	792 (46.2)	408 (59.7)	13 (17.6)	315 (44.2)	56 (22.7)
Secondary	809 (47.1)	225 (32.9)	50 (67.5)	356 (50.0)	178 (72.0)
Unknown	115 (6.7)	50 (7.4)	11 (14.9)	41 (5.8)	13 (5.3)
**Peru:**	**1,339 (78.0)**	**520 (38.8)**	**46 (3.4)**	**527 (39.4)**	**246 (18.4)**
La Merced	118 (8.9)	0 (0)	0 (0)	72 (13.6)	46 (18.6)
Puerto Maldonado	353 (26.4)	20 (3.9)	46 (100)	287 (54.5)	0 (0)
Piura	226 (16.8)	213 (40.9)	0 (0)	3 (0.6)	10 (4.1)
Tumbes	311 (23.2)	269 (51.7)	0 (0)	0 (0)	42 (17.1)
Yurimaguas	331 (24.7)	18 (3.5)	0 (0)	165 (31.3)	148 (60.2)
**Bolivia:**	**217 (12.7)**	**98 (45.2)**	**24 (11.0)**	**95 (43.8)**	**0 (0)**
Concepcion	51 (23.5)	36 (36.7)	3 (12.5)	12 (12.6)	0 (0)
Magdalena	1 (0.5)	0 (0)	0 (0)	1 (1.1)	0 (0)
Santa Cruz	89 (41.0)	49 (50.0)	21 (87.5)	19 (20.0)	0 (0)
Villa Tunari	76 (35.0)	13 (13.3)	0 (0)	63 (66.3)	0 (0)
**Ecuador:**	**26 (1.5)**	**13 (50.0)**	**0 (0)**	**12 (46.2)**	**1 (3.8)**
Guayaquil	17 (65.4)	4 (30.8)	0 (0)	12 (100)	1 (100)
Puyo	9 (34.6)	9 (69.2)	0 (0)	0 (0)	0 (0)
**Paraguay:**	**134 (7.8)**	**52 (38.8)**	**4 (3.0)**	**78 (58.2)**	**0 (0)**
Asuncion	43 (32.1)	9 (17.3)	2 (50.0)	32 (41.0)	0 (0)
Central	32 (23.9)	0 (0)	0 (0)	32 (41.0)	0 (0)
Ciudad de Este	50 (37.3)	42 (80.8)	2 (50.0)	6 (7.7)	0 (0)
Encarnacion	2 (1.5)	0 (0)	0 (0)	2 (2.6)	0 (0)
Filadelfia	7 (5.2)	1 (1.9)	0 (0)	6 (7.7)	0 (0)
Year of Diagnosis:					
2005	137 (7.9)	33 (4.8)	0 (0)	104 (14.6)	0 (0)
2006	94 (5.5)	21 (3.1)	1 (1.4)	70 (9.9)	2 (0.8)
2007	248 (14.4)	9 (1.3)	54 (72.9)	185 (25.9)	0 (0)
2008	142 (8.3)	29 (4.3)	1 (1.4)	61 (8.7)	51 (20.7)
2009	449 (26.2)	258 (37.7)	6 (8.1)	121 (16.9)	64 (25.9)
2010	646 (37.7)	333 (48.8)	12 (16.2)	171 (24.0)	130 (52.6)

Comparing the frequency of serotype-specific DENV infection by year of diagnosis, a majority of DENV-1 and DENV-4 infections occurred in 2009 and 2010, while a majority of DENV-2 infections occurred in 2007. Lastly, a higher prevalence of DENV-3 was observed between 2005–2007.

The majority of DENV-infected patients were from Peru (78.0%), followed by Bolivia (12.7%), Paraguay (7.8%), and Ecuador (1.5%). There was significant heterogeneity in the prevalence of dengue serotype by study site.

In Peru, Piura and Tumbes had the highest prevalence of DENV-1 as compared to other DENV serotypes (p<0.001 for comparison with other sites in Peru) while all DENV-2 infections and a majority of DENV-3 infections were detected in Puerto Maldonado. Additionally, Yurimaguas had the highest prevalence of DENV-4 as compared to other DENV serotypes (p = 0.002 for comparison with other sites in Peru). In Bolivia, Santa Cruz had the highest prevalence of DENV-1 and DENV-2 (p = 0.001 for both), and Villa Tunari had the highest prevalence of DENV-3 (p = 0.001). In Ecuador, all individuals infected with DENV-3 and DENV-4 were diagnosed in Guayaquil, and a higher proportion of individuals infected with DENV-1 were diagnosed in Puyo (p = 0.01). In Paraguay, a higher percentage of DENV-1 cases were diagnosed in Ciudad del Este (p = 0.002); furthermore, a higher percentage of DENV-3 infections were diagnosed in the two cities of Asuncion and Central, Paraguay (p = 0.01 for both).

Individuals with DENV-2 and DENV-4 infections were more likely to have been previously exposed to DENV (p<0.001 for both). Conversely, individuals with DENV-1 were less likely to have been previously exposed to DENV (p<0.001).

### Prevalence of Clinical Manifestation by DENV Serotype

#### Constitutional manifestations

A majority of individuals with DENV infection had manifestations in this category (99.5%) at the time of their clinic visit (**Tables 3** **and S1**). As compared to individuals infected with other DENV serotypes, individuals with DENV-1 were less likely to report malaise (p<0.001). As compared to individuals with DENV infection of other serotypes, individuals with DENV-2 were more likely to report malaise (p<0.001). As compared to individuals with DENV infection of other serotypes, individuals with DENV-3 had a higher prevalence of malaise (p<0.001), headache (p = 0.002), and prostration (p<0.001). Conversely, individuals with DENV-3 had a lower prevalence of retro-orbital pain (p = 0.001). Lastly, individuals with DENV-4, as compared to those with DENV infection of other serotypes, were less likely to report prostration (p<0.001).

**Table 3:** Association of constitutional, respiratory, gastrointestinal, and musculoskeletal manifestations and DENV serotype.

	Total*N(%)(N = 1,716)	DENV-1(n = 683) (39.8%)		DENV-2(n = 74) (4.3%)		DENV-3(n = 712) (41.5%)		DENV-4(n = 247) (14.4%)	
		InfectedN(%)	PR**95% CI	InfectedN(%)	PR**95% CI	InfectedN(%)	PR**(95% CI)	InfectedN(%)	PR**95% CI
**Constitutional**	**1,708 (99.5)**	**679 (99.4)**	**0.99 (0.99, 1.01)**	**74 (100.0)**	**1.00 (0.99, 1.01)**	**708 (99.4)**	**0.99 (0.99, 1.00)**	**247 (100.0)**	**1.00 (0.99, 1.01)**
Malaise	1,654 (96.4)	644 (94.3)	0.96 (0.94, 0.98)	74 (100.0)	1.03 (1.02, 1.04)	695 (97.6)	1.03 (1.01, 1.05)	241 (97.6)	1.01 (0.99, 1.04)
Headache	1,565 (91.2)	616 (90.2)	0.99 (0.96, 1.01)	58 (78.4)	0.89 (0.79, 1.00)	651 (91.4)	1.04 (1.01, 1.06)	240 (97.2)	0.99 (0.96, 1.01)
Retro-Orbital Pain	1,167 (68.0)	484 (70.9)	1.13 (1.05, 1.22)	43 (58.1)	0.88 (0.71, 1.09)	450 (63.2)	0.88 (0.81, 0.95)	190 (76.9)	1.04 (0.95, 1.13)
Prostration	695 (40.5)	245 (35.9)	0.83 (0.73, 0.95)	17 (22.9)	0.55 (0.35, 0.86)	373 (52.4)	1.66 (1.47, 1.89)	60 (24.3)	0.55 (0.43, 0.69)
**Respiratory**	**528 (30.8)**	**170 (24.9)**	**0.88 (0.75, 1.05)**	**25 (33.8)**	**0.83 (0.57, 1.22)**	**244 (34.3)**	**1.03 (0.88, 1.20)**	**89 (36.0)**	**1.26 (1.02, 1.56)**
Pharyngeal Congestion	394 (22.9)	125 (18.3)	0.93 (0.76, 1.13)	10 (13.5)	0.43 (0.23, 0.82)	181 (25.4)	0.98 (0.81, 1.17)	78 (31.6)	1.49 (1.17, 1.91)
Cough	207 (12.1)	80 (11.7)	0.97 (0.72, 1.30)	13 (17.6)	1.48 (0.81, 2.69)	87 (12.2)	1.01 (0.74, 1.37)	27 (10.9)	0.90 (0.59, 1.64)
Rhinorrhea	169 (9.9)	80 (11.7)	1.73 (1.28, 2.33)	4 (5.4)	0.42 (0.14, 1.27)	58 (8.2)	0.62 (0.46, 0.85)	27 (10.9)	1.06 (0.69, 1.64)
Dyspnea	23 (1.3)	3 (0.4)	0.21 (0.04, 0.98)	1 (1.4)	0.81 (0.11, 5.88)	17 (2.4)	4.34 (1.16, 16.3)	2 (0.8)	1.22 (0.26, 5.79)
Rhonchi	12 (0.7)	3 (0.4)	0.53 (0.09, 3.09)	1 (1.4)	3.24 (0.35, 29.6)	7 (0.9)	0.99 (0.19, 5.05)	1 (0.4)	1.50 (0.14, 16.1)
Wheezing	10 (0.6)	4 (0.6)	1.76 (0.51, 6.05)	1 (1.4)	3.68 (0.36, 38.0)	5 (0.7)	0.56 (0.14, 2.33)	0 (0)	-----
Cyanosis	3 (0.1)	1 (0.2)	1.86 (0.07, 51.3)	0 (0)	-----	2 (0.3)	1.18 (0.02, 59.3)	0 (0)	-----
**Gastrointestinal**	**1,204 (70.2)**	**457 (66.9)**	**0.90 (0.84, 0.96)**	**43 (58.1)**	**0.82 (0.66, 1.02)**	**536 (75.3)**	**1.25 (1.17, 1.33)**	**168 (68.0)**	**0.85 (0.77, 0.94)**
Nausea	981 (57.2)	364 (53.3)	0.88 (0.79, 0.96)	31 (41.9)	0.68 (0.49, 0.94)	456 (64.0)	1.37 (1.26, 1.49)	130 (52.6)	0.77 (0.67, 0.88)
Abdominal Pain	766 (44.6)	250 (36.6)	0.70 (0.62, 0.79)	25 (33.8)	0.69 (0.47, 1.02)	384 (53.9)	1.65 (1.48, 1.84)	107 (43.3)	0.79 (0.67, 0.94)
Vomiting	456 (26.6)	194 (28.4)	1.09 (0.91, 1.30)	12 (16.2)	0.51 (0.27, 0.98)	202 (28.4)	1.29 (1.08, 1.54)	48 (19.4)	0.58 (0.43, 0.78)
Diarrhea	296 (17.3)	104 (15.2)	0.67 (0.53, 0.84)	10 (13.5)	0.86 (0.45, 1.64)	153 (21.5)	1.96 (1.57, 2.44)	29 (11.7)	0.54 (0.37, 0.79)
Abdominal Distension	20 (1.2)	7 (1.0)	1.23 (0.37, 4.07)	1 (1.4)	1.06 (0.14, 7.75)	10 (1.4)	0.85 (0.23, 3.15)	2 (0.8)	1.37 (0.29, 6.49)
Hepatomegaly	20 (1.2)	5 (0.7)	0.49 (0.16, 1.54)	0 (0)	-----	11 (1.5)	2.07 (0.83, 5.16)	4 (1.6)	1.20 (0.32, 4.50)
Jaundice	10 (0.6)	5 (0.7)	1.17 (0.24, 5.64)	1 (1.4)	2.90 (0.33, 25.7)	3 (0.4)	0.52 (0.10, 2.63)	1 (0.4)	1.35 (0.17, 10.8)
Splenomegaly	7 (0.4)	1 (0.2)	0.32 (0.03, 3.42)	0 (0)	-----	4 (0.6)	2.64 (0.43, 16.1)	2 (0.8)	1.39 (0.12, 16.0)
Ascites	4 (0.2)	2 (0.3)	1.52 (0.18, 12.9)	0 (0)	-----	2 (0.3)	1.09 (0.13, 8.83)	0 (0)	-----
**Musculoskeletal**	**1,619 (94.3)**	**645 (94.4)**	**1.00 (0.98, 1.02)**	**62 (83.8)**	**0.94 (0.85, 1.02)**	**677 (95.1)**	**1.02 (1.00, 1.04)**	**235 (95.1)**	**0.98 (0.95, 1.01)**
Myalgia	1,546 (90.1)	613 (89.8)	0.99 (0.96, 1.03)	52 (70.3)	0.80 (0.68, 0.93)	653 (91.7)	1.05 (1.02, 1.08)	228 (92.3)	0.98 (0.94, 1.02)
Bone Pain	1,279 (74.5)	565 (82.7)	1.22 (1.15, 1.30)	43 (58.1)	0.87 (0.73, 1.05)	523 (73.5)	0.96 (0.89, 1.02)	148 (59.9)	0.76 (0.68, 0.85)
Joint Pain†	1,165 (67.9)	359 (52.6)	0.67 (0.61, 0.73)	51 (68.9)	1.04 (0.89, 1.20)	579 (81.3)	1.39 (1.29, 1.49)	176 (71.3)	1.07 (0.98, 1.18)
**Cutaneous**	**377 (21.9)**	**157 (22.9)**	**1.17 (0.97, 1.42)**	**5 (6.8)**	**0.34 (0.15, 0.81)**	**104 (14.6)**	**0.49 (0.40, 0.61)**	**111 (44.9)**	**2.34 (1.91, 2.86)**
Central Erythema	305 (17.8)	122 (17.9)	0.98 (0.79, 1.19)	2 (2.7)	0.21 (0.05, 0.85)	73 (10.3)	0.51 (0.39, 0.65)	108 (43.7)	2.58 (2.07, 3.21)
Distal Erythema	281 (16.4)	104 (15.2)	0.87 (0.70, 1.09)	3 (4.1)	0.33 (0.11, 1.02)	81 (11.4)	0.67 (0.53, 0.85)	93 (37.7)	2.18 (1.71, 2.79)
Facial Erythema	217 (12.7)	81 (11.9)	0.87 (0.67, 1.13)	1 (1.4)	0.15 (0.02, 1.12)	54 (7.6)	0.47 (0.35, 0.63)	81 (32.8)	3.39 (2.57, 4.46)
Maculopapular Rash	128 (7.5)	42 (6.2)	0.99 (0.67, 1.46)	1 (1.4)	0.13 (0.02, 0.92)	47 (6.6)	0.58 (0.39, 0.84)	38 (15.4)	3.18 (2.01, 5.03)
Vesicles	8 (0.5)	2 (0.3)	0.30 (0.03, 2.99)	0 (0)	-----	5 (0.7)	2.28 (0.39, 13.0)	1 (0.4)	2.83 (0.18, 44.5)
Subcutaneous Nodules	6 (0.4)	1 (0.2)	0.91 (0.10, 7.96)	0 (0)	-----	5 (0.7)	2.88 (0.24, 33.8)	0 (0)	-----
**Neurological**	**47 (2.7)**	**8 (1.2)**	**0.44 (0.16, 1.18)**	**8 (10.8)**	**3.02 (1.06, 8.58)**	**31 (4.4)**	**1.83 (0.72, 4.65)**	**0 (0)**	**-----**
Impaired Mental Status	26 (1.5)	4 (0.6)	0.56 (0.17, 1.79)	4 (5.4)	3.93 (1.10, 14.0)	18 (2.5)	1.35 (0.43, 4.24)	0 (0)	-----
Neck Stiffness	20 (1.2)	4 (0.6)	0.27 (0.03, 2.29)	4 (5.4)	2.14 (0.28, 16.5)	12 (1.7)	2.84 (0.43, 18.6)	0 (0)	-----
Seizures	6 (0.4)	1 (0.2)	0.42 (0.04, 4.25)	0 (0)	-----	5 (0.7)	5.72 (0.49, 65.8)	0 (0)	-----
Focal Signs	1 (0.1)	0 (0)	-----	0 (0)	-----	1 (0.1)	1.00 (0.99, 1.01)	0 (0)	-----

The reference group for each Prevalence Ratio (PR) associated with a given DENV serotype group are the other three DENV serotype groups.

*: Total number of individuals presenting with a given sign or symptom.

**: All prevalence ratios adjusted for age, sex, location, day of illness, immune status (primary vs secondary), and year of diagnosis.

**†:** Includes those with arthralgia, arthritis, or joint incapacity.

#### Respiratory manifestations

The prevalence of respiratory manifestations in this sample was 30.8% (**Tables 3** **and S1**). Individuals infected with DENV-1 had a higher prevalence of rhinorrhea compared to individuals infected with other DENV serotypes (p<0.001). Individuals infected with DENV-3 had a lower prevalence of rhinorrhea (p = 0.003) compared to individuals infected with other DENV serotypes. Individuals infected with DENV-4 had a higher prevalence of pharyngeal congestion (p = 0.001) compared to individuals infected with other DENV serotypes.

#### Gastrointestinal manifestations

Greater than half of the participants had gastrointestinal manifestations (70.2%) (**Tables 3** **and S1**). Compared to individuals infected with other DENV serotypes, a lower prevalence of gastrointestinal manifestations was found in those infected with DENV-1 (p = 0.003), characterized by a lower prevalence of abdominal pain (p<0.001) and diarrhea (p = 0.001). Relative to those infected with other DENV serotypes, individuals infected with DENV-3 were more likely to report gastrointestinal manifestations (p<0.001), characterized by a higher prevalence of nausea (p<0.001), abdominal pain (p<0.001), vomiting (p = 0.005), and diarrhea (p<0.001). Among those infected with DENV-4, a lower prevalence of gastrointestinal manifestations in general (p = 0.001) characterized by a lower prevalence of nausea (p = 0.001), vomiting (p<0.001), and diarrhea (p = 0.002) were found compared to those infected with the other three DENV serotypes.

#### Musculoskeletal manifestations

Musculoskeletal manifestations were reported by a majority of individuals in this sample (94.3%) (**Tables 3** **and S1**). Individuals with DENV-1 were more likely to report bone pain (p<0.001), but less likely to report joint pain (p<0.001) compared with those infected with other DENV serotypes. Individuals with DENV-2 were less likely to report myalgia (p = 0.004) than those infected with other DENV serotypes. Compared with those infected with other DENV serotypes, individuals infected with DENV-3 were more likely to report joint paint (p<0.001) and myalgia (p = 0.001). Lastly, individuals with DENV-4 were less likely to report bone pain (p<0.001) compared to those infected with other DENV serotypes.

#### Cutaneous manifestations

Cutaneous manifestations were reported by 21.9% of the sample (**Tables 3** **and S1**). Individuals with DENV-3 were less likely to have cutaneous manifestations (p<0.001) when compared to those infected with other serotypes. The lower prevalence of cutaneous manifestations among those with DENV-3 was characterized by a lower prevalence of central erythema (p<0.001), facial erythema (p<0.001), distal erythema (p = 0.001), and maculopapular rash (p = 0.004). Conversely, compared with those infected with other DENV serotypes, the prevalence of cutaneous manifestations was higher among individuals with DENV-4 (p<0.001) and was characterized by a higher prevalence of central erythema (p<0.001), distal erythema (p<0.001), facial erythema (p<0.001), and maculopapular rash (p<0.001).

#### Neurological manifestations

Neurological manifestations were reported by 2.7% of the sample (**Tables 3** **and S1**). No difference in neurological manifestations was observed by DENV serotype.

## Discussion

This cross-sectional study, conducted as part of an on-going passive surveillance screening program across a wide geographic region of western South America, identified distinct differences in the prevalence of clinical manifestations by DENV serotype. Individuals with DENV-3 had a higher prevalence of musculoskeletal and gastrointestinal manifestations, whereas individuals with DENV-4 infection had a higher prevalence of cutaneous and respiratory manifestations. The recent introduction and establishment of multiple co-circulating serotypes of DENV in various regions of the world, including South America, has heightened the importance of understanding and characterizing the role each serotype plays in the clinical outcome of infection.

Constitutional manifestations are well-known to DENV infection, but are also present in many other diseases. Unlike any study to date, our study uniquely demonstrated a significantly higher frequency of headache and prostration with DENV-3 infection when compared with infection with the other DENV serotypes. In addition, the higher prevalence of malaise with DENV-2 and DENV-3 compared with the other DENV serotypes was also a novel finding.

DENVs have been detected and isolated in respiratory specimens of both the upper and lower respiratory tract from individuals with confirmed dengue fever or DHF [27], [28]. Only a handful of studies have examined DENV serotype differences in respiratory manifestations. One study from Thailand [19] noted no difference between all four serotypes when assessing for the presence of pleural effusion, although a higher pleural effusion index was found in DENV-2 compared with DENV-1. Our study did not specifically examine pleural effusions. A study from Taiwan [14] comparing only two DENV serotypes showed a common respiratory endpoint (combining cough, rhinorrhea, nasal stuffiness, or sore throat) was more prevalent in DENV-3 than DENV-2. In contrast, we looked at all four DENV serotypes and found two respiratory correlations not reported previously in the literature: an increased prevalence of rhinorrhea with DENV-1 infection and an increased prevalence of pharyngeal congestion with DENV-4 infection.

DENV infection can result in a wide array of gastrointestinal manifestations and can often be mistaken for other entities, including acute appendicitis, acute cholecystitis, and diffuse peritonitis [29], [30]. Most previous studies examining the common symptoms associated with gastrointestinal illness such as nausea, vomiting, diarrhea, and abdominal pain showed no association with specific dengue serotype [13], [15], [16]. However, a study from India [22] with all four DENV serotypes demonstrated more abdominal pain in those infected with DENV2 and one study from Martinique with only two serotypes noted a higher prevalence of unspecified gastrointestinal manifestations among those with DENV-2 as compared to DENV-4 [17]. Our findings of a higher prevalence of gastrointestinal manifestations, specifically symptoms such as nausea, abdominal pain, vomiting and diarrhea among those infected with DENV-3 compared to the other three DENV serotypes has not been reported prior to this. Regarding gastrointestinal signs, a pediatric study from Thailand [20] reported a higher prevalence of hepatomegaly among those infected with DENV-2 and DENV-3 and a study from India [22] found a higher prevalence with DENV-2, whereas others studies [13], [15] found no difference between DENV serotypes for this sign. Two prior studies [14], [19] examining ascites observed a higher prevalence among individuals with DENV-3 (compared to DENV-2) and DENV-2 (compared to the other three DENV serotypes). In our population, we did not find any correlation between a specific DENV serotype and the signs of abdominal distension, hepatomegaly, splenomegaly, jaundice, or ascites.

DF is nicknamed “breakbone fever” and it was therefore not surprising that the three individual constituents of the “musculoskeletal manifestation” category–muscle, bone, and joint pains–were each reported by over two-thirds of all participants surveyed. Our statistically significant finding of DENV-3 causing more myalgia was also found in a study from Taiwan [14] that examined only two serotypes (DENV-2 and DENV-3). In addition, we found that infection with DENV-3 predisposed to more joint pain, a novel finding. In comparison, a study from Nicaragua [18] demonstrated more arthralgia in infection with DENV-2 than DENV-1. A study from another group in Peru [16] found significantly different amounts of bone pain in the three serotypes they investigated, with DENV-2 having the most, DENV-1 having the second most, and DENV-3 having the least. This is in contrast with our results, which showed a greater percentage of bone pain with DENV-1 infection.

Skin findings associated with DENV infection are well-described [31] and may be useful in distinguishing DENV infection from other endemic causes of febrile disease [32]. Classically, flushing or a macular erythematous rash affecting the face, neck, and chest is noted within the first 48 hours of symptom onset, later evolving to a more maculopapular rash. Individuals infected with DENV-4 had a significantly higher prevalence of cutaneous manifestations as compared to individuals infected with any of the other DENV serotypes in this study. Furthermore, this elevated prevalence in DENV-4 infection was observed for the specific cutaneous findings of erythematous rash across all locations (facial, central, or distal) as well as maculopapular rash, all of which are unreported findings. A Taiwanese study of two DENV serotypes found that those infected with DENV-3 were more likely to have a rash than those infected with DENV-2 (51.6% vs 29.2%) [14], a trend we also found with those two serotypes (14.6% vs 6.8%; p<0.01). A study from another population in Peru noted a greater percentage of non-petechiael, non-ecchymotic rash in participants infected with DENV-1 (23.1%) compared to DENV-2 (14.6%) or DENV-3 (7.1%) [16]. Our results also demonstrated a similar higher prevalence of these manifestations in DENV-1 as compared to DENV-2 or DENV-3.

Neurological findings such as neck stiffness, impaired mental status, focal motor deficits, and seizures have been described primarily in individual reports or small case series and the majority have been found to be associated with either DENV-2 or DENV-3 infection [15], [33]. Although not statistically significant, we also noted a higher proportion of neurological findings with DENV-2 and DENV-3.

This study has some limitations. First, our utilization of a clinic-based passive surveillance system, compared with hospital-based surveillance, most likely sampled patients with less severe disease. Conversely, compared with home-visit-based active surveillance, our study most likely recruited patients with more severe disease. Such differences in sampling strategies are not necessarily a weakness, but merit attention when considering the results. Nevertheless, according to the Pan American Health Organization, the annual ratio of DHF (or severe) cases to total dengue cases in the four countries during the study period was extremely low, most often numbering fewer than 1 in 500 [34]. Second, our study was not designed to collect ancillary laboratory data (i.e., hematocrit, platelet count, and total protein) that would have helped fulfill criteria for DHF classification [35]. For the reasons outlined above, our study recruited only patients with dengue fever and not DHF. While we did observe differences in certain clinical manifestations by DENV serotype, we still cannot rule out the possibility of underlying co-morbidities driving a particular manifestation clustering within a DENV serotype. Last, given the cross-sectional nature of this study, the differences in prevalence of signs and symptoms by DENV serotype are purely corollary, requiring additional, longitudinal studies to better assess both the temporality of DENV infection with manifestation occurrence as well as the specificity of the relationship.

While much of the research on signs and symptoms has focused on the temporal order of DENV infection [8], [36] or the unique pathogenic role of DENV-2 [10], [37] on outcomes such as DHF, this is one of the first studies to perform a comprehensive evaluation of clinical manifestations across all four DENV serotypes in the Americas. The use of a highly specific laboratory assay such as IFA to detect and serotype every DENV in conjunction with standardized reporting and collection of clinical information across a number of geographically diverse settings in South America limits the potential for misclassification and other potential biases. Our results indicate that specific individual manifestations, as well as certain manifestation groups, were often over-represented by a specific DENV serotype, emphasizing the need to consider infection with each serotype as a distinct clinical entity. More large-scale cross-sectional studies, as well as longitudinal studies associating the temporal order of serotype-specific DENV infection and the development of clinical manifestations, are needed to confirm some of the novel findings of this study. In addition, future studies concentrating on clinical differences within serotypes (e.g., genotypes and lineages of a certain serotype) will further elucidate the role between individual DENV serotypes and morbidity.

## Supporting Information

Table S1 P-values for the difference in sign/symptom prevalence by DENV serotype.
(DOCX)
