# Supplementary material for: Correlation of Serotype-Specific Dengue Virus Infection with Clinical Manifestations
Source: PLoS Negl Trop Dis. 2012 May 1;6(5):e1638. doi: 10.1371/journal.pntd.0001638 (PMC3341333; doi:10.1371/journal.pntd.0001638)
Supplement: Table S1 — P-values for the difference in sign/symptom prevalence by DENV serotype. (DOCX) [file pntd.0001638.s001.docx]

|  | **Total***  **N(%)**  **(N=1,716)** | **DENV-1**  **(n=683) (39.8%)** | | **DENV-2**  **(n=74) (4.3%)** | | **DENV-3**  **(n=712) (41.5%)** | | **DENV-4**  **(n=247) (14.4%)** | |
| --- | --- | --- | --- | --- | --- | --- | --- | --- | --- |
|  |  | **Infected**  **N(%)** | **P-value** | **Infected**  **N(%)** | **p-value** | **Infected**  **N(%)** | **p-value** | **Infected**  **N(%)** | **p-value** |
| **Constitutional** | **1,708 (99.5)** | **679 (99.4)** | **0.784** | **74 (100.0)** | **0.145** | **708 (99.4)** | **0.522** | **247 (100.0)** | **0.484** |
| Malaise | 1,654 (96.4) | 644 (94.3) | <0.001 | 74 (100.0) | <0.001 | 695 (97.6) | <0.001 | 241 (97.6) | 0.391 |
| Headache | 1,565 (91.2) | 616 (90.2) | 0.358 | 58 (78.4) | 0.054 | 651 (91.4) | 0.002 | 240 (97.2) | 0.355 |
| Retro-Orbital Pain | 1,167 (68.0) | 484 (70.9) | 0.001 | 43 (58.1) | 0.228 | 450 (63.2) | 0.001 | 190 (76.9) | 0.366 |
| Prostration | 695 (40.5) | 245 (35.9) | 0.007 | 17 (22.9) | 0.009 | 373 (52.4) | <0.001 | 60 (24.3) | <0.001 |
|  |  |  |  |  |  |  |  |  |  |
| **Respiratory** | **528 (30.8)** | **170 (24.9)** | **0.101** | **25 (33.8)** | **0.292** | **244 (34.3)** | **0.579** | **89 (36.0)** | **0.032** |
| Pharyngeal Congestion | 394 (22.9) | 125 (18.3) | 0.452 | 10 (13.5) | 0.011 | 181 (25.4) | 0.801 | 78 (31.6) | 0.001 |
| Cough | 207 (12.1) | 80 (11.7) | 0.827 | 13 (17.6) | 0.202 | 87 (12.2) | 0.973 | 27 (10.9) | 0.637 |
| Rhinorrhea | 169 (9.9) | 80 (11.7) | <0.001 | 4 (5.4) | 0.125 | 58 (8.2) | 0.003 | 27 (10.9) | 0.777 |
| Dyspnea | 23 (1.3) | 3 (0.4) | 0.047 | 1 (1.4) | 0.832 | 17 (2.4) | 0.029 | 2 (0.8) | 0.799 |
| Rhonchi | 12 (0.7) | 3 (0.4) | 0.479 | 1 (1.4) | 0.297 | 7 (0.9) | 0.998 | 1 (0.4) | 0.737 |
| Wheezing | 10 (0.6) | 4 (0.6) | 0.372 | 1 (1.4) | 0.274 | 5 (0.7) | 0.428 | 0 (0) | ----- |
| Cyanosis | 3 (0.1) | 1 (0.2) | 0.714 | 0 (0) | ----- | 2 (0.3) | 0.925 | 0 (0) | ----- |
|  |  |  |  |  |  |  |  |  |  |
| **Gastrointestinal** | **1,204 (70.2)** | **457 (66.9)** | **0.003** | **43 (58.1)** | **0.097** | **536 (75.3)** | **<0.001** | **168 (68.0)** | **0.001** |
| Nausea | 981 (57.2) | 364 (53.3) | 0.006 | 31 (41.9) | 0.018 | 456 (64.0) | <0.001 | 130 (52.6) | <0.001 |
| Abdominal Pain | 766 (44.6) | 250 (36.6) | <0.001 | 25 (33.8) | 0.062 | 384 (53.9) | <0.001 | 107 (43.3) | 0.007 |
| Vomiting | 456 (26.6) | 194 (28.4) | 0.349 | 12 (16.2) | 0.044 | 202 (28.4) | 0.005 | 48 (19.4) | <0.001 |
| Diarrhea | 296 (17.3) | 104 (15.2) | 0.001 | 10 (13.5) | 0.647 | 153 (21.5) | <0.001 | 29 (11.7) | 0.002 |
| Abdominal Distension | 20 (1.2) | 7 (1.0) | 0.737 | 1 (1.4) | 0.955 | 10 (1.4) | 0.803 | 2 (0.8) | 0.691 |
| Hepatomegaly | 20 (1.2) | 5 (0.7) | 0.226 | 0 (0) | ----- | 11 (1.5) | 0.119 | 4 (1.6) | 0.790 |
| Jaundice | 10 (0.6) | 5 (0.7) | 0.845 | 1 (1.4) | 0.338 | 3 (0.4) | 0.432 | 1 (0.4) | 0.775 |
| Splenomegaly | 7 (0.4) | 1 (0.2) | 0.344 | 0 (0) | ----- | 4 (0.6) | 0.293 | 2 (0.8) | 0.789 |
| Ascites | 4 (0.2) | 2 (0.3) | 0.701 | 0 (0) | ----- | 2 (0.3) | 0.939 | 0 (0) | ----- |
|  |  |  |  |  |  |  |  |  |  |
| **Musculoskeletal** | **1,619 (94.3)** | **645 (94.4)** | **0.825** | **62 (83.8)** | **0.159** | **677 (95.1)** | **0.029** | **235 (95.1)** | **0.077** |
| Myalgia | 1,546 (90.1) | 613 (89.8) | 0.869 | 52 (70.3) | 0.004 | 653 (91.7) | 0.001 | 228 (92.3) | 0.256 |
| Bone Pain | 1,279 (74.5) | 565 (82.7) | <0.001 | 43 (58.1) | 0.147 | 523 (73.5) | 0.202 | 148 (59.9) | <0.001 |
| Joint Pain† | 1,165 (67.9) | 359 (52.6) | <0.001 | 51 (68.9) | 0.638 | 579 (81.3) | <0.001 | 176 (71.3) | 0.143 |
|  |  |  |  |  |  |  |  |  |  |
| **Cutaneous** | **377 (21.9)** | **157 (22.9)** | **0.096** | **5 (6.8)** | **0.014** | **104 (14.6)** | **<0.001** | **111 (44.9)** | **<0.001** |
| Central Erythema | 305 (17.8) | 122 (17.9) | 0.826 | 2 (2.7) | 0.028 | 73 (10.3) | <0.001 | 108 (43.7) | <0.001 |
| Distal Erythema | 281 (16.4) | 104 (15.2) | 0.229 | 3 (4.1) | 0.054 | 81 (11.4) | 0.001 | 93 (37.7) | <0.001 |
| Facial Erythema | 217 (12.7) | 81 (11.9) | 0.310 | 1 (1.4) | 0.041 | 54 (7.6) | <0.001 | 81 (32.8) | <0.001 |
| Maculopapular Rash | 128 (7.5) | 42 (6.2) | 0.963 | 1 (1.4) | 0.065 | 47 (6.6) | 0.004 | 38 (15.4) | <0.001 |
| Vesicles | 8 (0.5) | 2 (0.3) | 0.302 | 0 (0) | ----- | 5 (0.7) | 0.354 | 1 (0.4) | 0.459 |
| Subcutaneous Nodules | 6 (0.4) | 1 (0.2) | 0.931 | 0 (0) | ----- | 5 (0.7) | 0.401 | 0 (0) | ----- |
|  |  |  |  |  |  |  |  |  |  |
| **Neurological** | **47 (2.7)** | **8 (1.2)** | **0.103** | **8 (10.8)** | **0.039** | **31 (4.4)** | **0.203** | **0 (0)** | **-----** |
| Impaired Mental Status | 26 (1.5) | 4 (0.6) | 0.327 | 4 (5.4) | 0.035 | 18 (2.5) | 0.603 | 0 (0) | ----- |
| Neck Stiffness | 20 (1.2) | 4 (0.6) | 0.229 | 4 (5.4) | 0.466 | 12 (1.7) | 0.276 | 0 (0) | ----- |
| Seizures | 6 (0.4) | 1 (0.2) | 0.459 | 0 (0) | ----- | 5 (0.7) | 0.162 | 0 (0) | ----- |
| Focal Signs | 1 (0.1) | 0 (0) | ----- | 0 (0) | ----- | 1 (0.1) | 0.999 | 0 (0) | ----- |
| The reference group for each PR associated with a given DENV serotype group are the other three DENV serotype groups  *Total number of individuals presenting with a given sign or symptom  **All prevalence ratios adjusted for age, sex, location, day of illness, immune status (primary vs secondary), and year of diagnosis  † Includes those with arthralgia, arthritis, or joint incapacity | | | | | | | | | |
